# Trichilemmal Cyst of the Eyelid: Masquerading as Recurrent Chalazion

**DOI:** 10.1155/2012/261414

**Published:** 2012-04-18

**Authors:** Manju Meena, Ruchi Mittal, Debarati Saha

**Affiliations:** ^1^Department of Ophthalmic Plastics, Facial Aesthetics and Ocular Oncology, GMRV Campus, L V Prasad Eye Institute, Hanumanthwaka Junction, Visakhapatnam, Andhra Pradesh 530040, India; ^2^Dalmia Ophthalmic Pathology Services, L V Prasada Eye Institute, Patia, Bhubaneswar, Orrisa 751024, India

## Abstract

A 52-years-old female presented with a history of a painless, progressive swelling in the left lower eyelid of one-year duration. The lesion was excised twice as a chalazion and recurred. Excisional biopsy of the mass was performed and histopathological findings were consistent with those of trichilemmal cyst. We report a rare case of trichilemmal cyst of the eyelid which was masquerading as chalazion for which the patient had undergone multiple surgeries. Wide excision was done and diagnosis was confirmed on histopathology. There was no recurrence seen till 2 months of postoperative period. Trichilemmal cyst, although rare, should be considered as differential diagnosis of recurrent chalazion.

## 1. Introduction

Trichilemmal cyst, also known as a pilar cyst, forms from the outer root sheath of hair follicle. They are common benign tumors most often found on the scalp [1]. The involvement of the eyelids is quite rare. It presents as a smooth, firm, mobile, and round nodule without a visible punctum. There is often an autosomal dominant inheritance pattern as the lesion is frequently familial [2]. Till now, there is only one case report of trichilemmal cyst of the eyelid [3]. To the best of our knowledge, this is the first case report of trichilemmal cyst masquerading as recurrent chalazion.

## 2. Case Report

**Figure 1:**
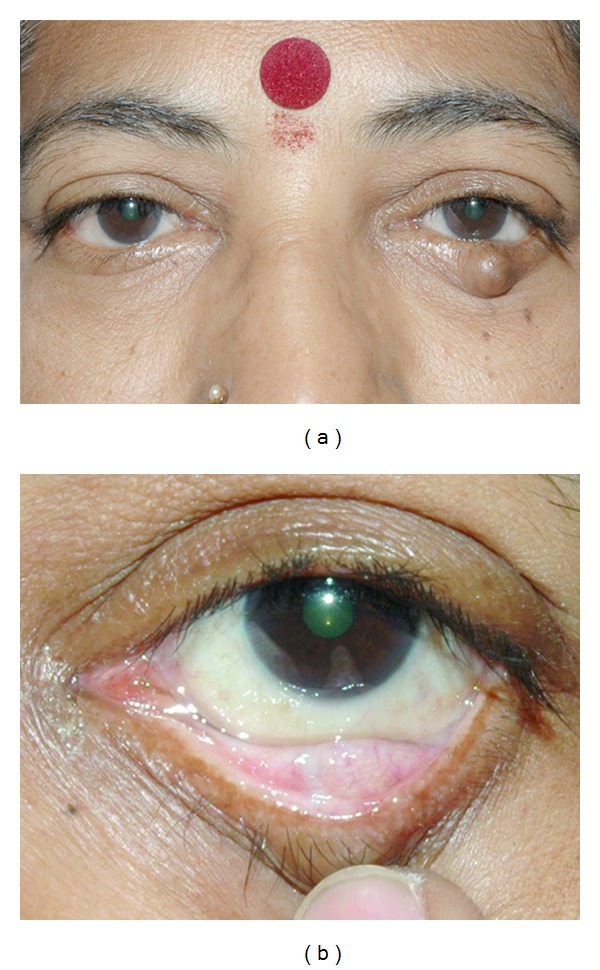
Figures 1(a) and 1(b) Showing a nodular mass measuring 10 × 10 × 8 mm in the lower eyelid with smooth surface. On eversion of the eyelid there was an area of scarring in the tarsal conjunctiva.

A 52-year-old lady presented with history of gradually progressing, painless swelling of the left lower eyelid of 1 year duration. She was diagnosed as chalazion elsewhere for which she had undergone incision and curettage twice following which the swelling recurred to the present size. There was a history of gradual increase in the size of the swelling following incision and curettage. There was no history of similar lesion anywhere else in the body. There was no significant family history. On clinical examination there was a firm, nodular mass measuring 10 × 10 × 8 mm (Figure 1(a)). The mass was fixed to the underlying tarsus, nontender and nonmobile. On eversion of the eyelid there was an area of scarring in the tarsal conjunctiva and the overlying skin was normal (Figure 1(b)). The examination of rest of the anterior and posterior segment was unremarkable. The right eye was essentially normal and systemic examination revealed normal findings. Full thickness excision biopsy was done under local anesthesia and eyelid defect was repaired by Tenzel's semicircular flap. The specimen was sent for histopathological examination.

**Figure 2:**
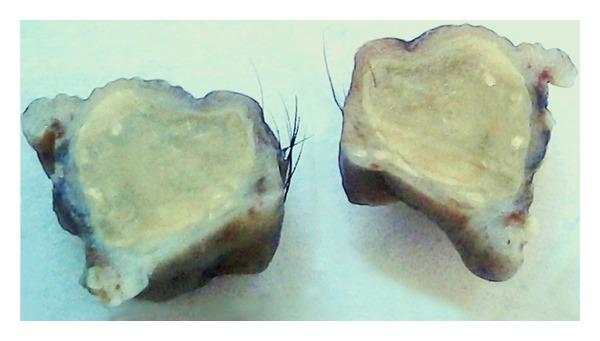
Gross photograph of full thickness pentagonal excision of eyelid shows well circumscribed cyst filled with solid, homogenous material with spotty greyish white chalky areas.

On gross examination, it was a pentagonal full-thickness segment of the eyelid measuring 11 × 9 × 9 mm consisted of a nodular mass lesion with smooth overlying surface. Cut section revealed a unilocular cyst measuring 9 mm in diameter. Cyst was filled with yellowish homogenous solid material with focal gritty white areas (Figure 2).

**Figure 3:**
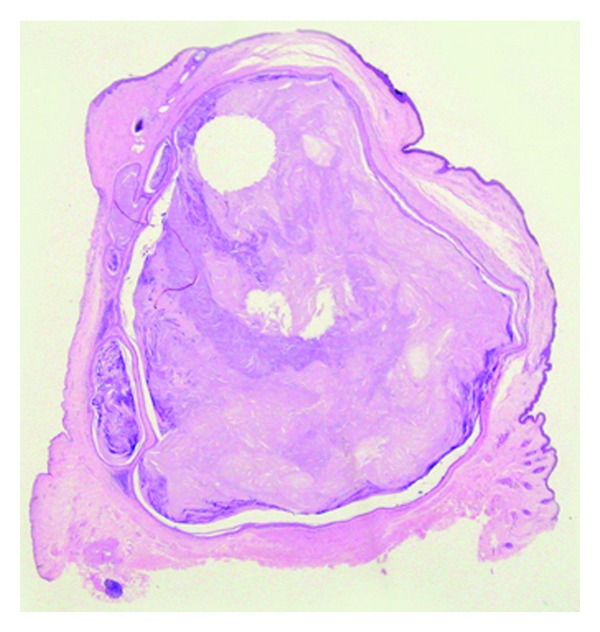
Whole mount photograph of lesion shown in Figure 1. Eyelid cyst filled with homogenous keratin.

**Figure 4:**
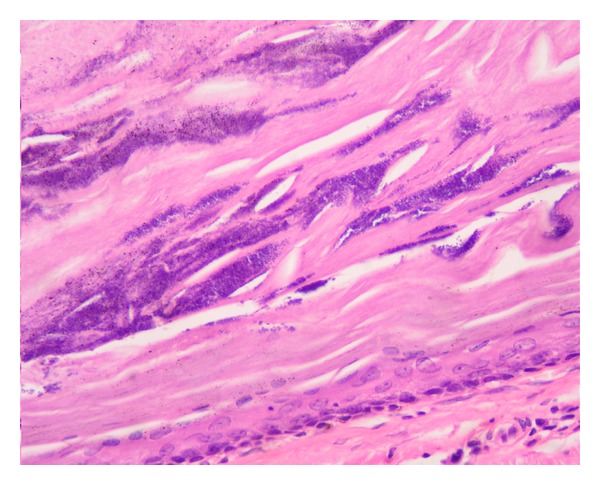
Histopathology of lesion shown in Figure 1. The epithelial lining below shows peripheral palisading of the peripheral cells. Inner cells are swollen. Cyst cavity is filled with homogenous keratin with calcification. (Hematoxylin-eosin ×400).

Microscopic examination showed lining keratinized stratified squamous epithelium of eyelid skin. Subepithelium showed a cyst, wall of which was composed of epithelial cells with absence of clearly visible intercellular bridges. The epithelial cells close to the cystic cavity appeared swollen filled with pale cytoplasm. The peripheral layer of cells showed distinct palisade arrangement. Cyst was filled with homogeneous, amorphous keratinous material. Patchy calcification of this keratinous material was noted. Cholesterol clefts were seen at places in the amorphous material (Figures 3 and 4).

A diagnosis of trichilemmal cyst of the eyelid was made based on histopathological findings. The postoperative period was uneventful and there was no recurrence of the swelling till 2 months following surgery.

## 3. Discussion

There are several types of benign and malignant lesions of the eyelids. The common benign lesions are chalazion, epidermal inclusion cysts, seborrheic keratosis, and apocrine hidrocystomas [4]. Trichilemmal cyst occurs more commonly on the hair bearing areas of the body with abundant hair follicles like scalp [1]. The involvement of other locations have also been reported [5]. Trichilemmal cyst were previously considered as sebaceous or epidermoid cysts and were historically referred to by the common name of Wen [5]. The name was changed when it became apparent that the keratinization in them is analogous to the keratinization that takes place in the outer root sheath of hair or trichilemma. Both trichilemmal and epidermal cyst are keratinous cysts and usually have similar clinical presentation [6]. However, these cysts differ significantly in their manner of keratinization on histopathology. Trichilemmal cyst shows trichilemmal or abrupt keratinization without keratohyalin granules. The peripheral layers demonstrate a palisading arrangement, whereas cells close to the cyst cavity are swollen and filled with pale cytoplasm. The cyst cavity contains amorphous eosinophilic keratin. Foci of calcifications within the keratin occur in approximately 25% of cases [7]. Therefore, histopathological examination is important to differentiate between the two conditions. Approximately 20% of epithelial cysts are trichilemmal cyst and 80% are epidermal [8]. Trichilemmal cyst is a benign lesion however rarely, these cysts may grow more extensively and form proliferating trichilemmal tumors, also called proliferating trichilemmal cysts, which are benign but may grow aggressively at the cyst site [9]. Very rarely, trichilemmal cysts and proliferating trichilemmal tumors can undergo malignant transformation [10]. Till now, there is only single case report of the eyelid involvement by the trichilemmal cyst [3]. In our case, before presentation the patient had undergone multiple surgeries for chalazion but the lesion recurred. The possible explanation could be the incomplete removal of the contents of the cyst without removing the cyst wall. Therefore recollection of the keratinous material in the cyst cavity would have led to the recurrence. Complete removal of the cyst along with the cyst wall is therefore mandatory in such cases followed by histopathological confirmation of the diagnosis. The uniqueness of our case is the clinical presentation as a chalazion which was different from the lesion described in the previous case report, fixity to the tarsus, multiple recurrence of the lesion at the same location following incision, and curettage procedure. To the best of our knowledge, this is the first case report which highlights the need for considering the trichilemmal cyst as a differential diagnosis of recurrent chalazion.
